# Enhancing Polydimethylsiloxane with Silver Nanoparticles for Biomedical Coatings

**DOI:** 10.3390/biomimetics10120846

**Published:** 2025-12-17

**Authors:** Axel Bachoux, Cédric Desroches, Laurence Bois, Catherine Journet, Aurore Berthier, Frédérique Bessueille-Barbier, Bérangère Toury, Nina Attik

**Affiliations:** 1 LMI UMR 5615, CNRS, Universite Claude Bernard Lyon 1, 69100 Villeurbanne, France; axel.bachoux@gmail.com (A.B.); cedric.desroches@univ-lyon1.fr (C.D.); laurence.bois@univ-lyon1.fr (L.B.); catherine.gautier@univ-lyon1.fr (C.J.); berangere.toury-pierre@univ-lyon1.fr (B.T.); 2 Laboratoire de Biologie Tissulaire et Ingénierie Thérapeutique—UMR 5305, Universite Lyon 1, CNRSL, Lyon Cedex 7, 69367 Lyon, France; aurore.berthier@cnrs.fr; 3 ISA UMR 5280, Institut des Sciences Analytiques, Université Claude Bernard Lyon 1, Université de Lyon, 69100 Villeurbanne, France; frederique.bessueille@isa-lyon.fr; 4 Faculté d’Odontologie de Lyon, Université Claude Bernard Lyon 1, 7 rue Guillaume Paradin, 69372 Lyon Cedex 08, France

**Keywords:** antibacterial activity, biomedical applications, coating, cytocompatibility, PDMS, silver nanoparticles

## Abstract

Silver nanoparticles (AgNPs) are widely used as antibacterial agents either as colloidal solutions or deposited on surfaces. However, the high concentration of AgNPs can lead to cytotoxicity, posing a hazard to healthy cells and tissues. Achieving a balance between antibacterial efficacy and cytocompatibility is crucial for biomedical applications. Polymeric coatings, especially those made from polydimethylsiloxane (PDMS) like Sylgard 184, are popular in biomedical applications due to their user-friendliness. We have developed a cost-effective method to reduce silver ions using the Si-H silane functions of PDMS in situ. Tetrahydrofuran (THF) acts as a solvent, inducing a swelling effect in PDMS, allowing silver ions from silver tetrafluoroborate (AgBF_4_) dissolved in THF to diffuse into the polymer and undergo reduction. This process results in PDMS functionalized with well-distributed 10 nm silver AgNPs. The resulting metal–polymer nanocomposites (MPNs) exhibit yellow shades and, based on qualitative Live/Dead staining observations, show no apparent cytotoxicity on human gingival fibroblasts. In addition, SEM analyses indicate a qualitative reduction in *E. coli* adhesion, suggesting an antibacterial anti-adhesive potential against this bacterial strain. Further studies should investigate the release profile of AgNPs in these composites, which could guide the development of new biocompatible coatings for phototherapy devices and enhance their long-term clinical performance.

## 1. Introduction

The increasing prevalence of implant-associated infections and antibiotic-resistant pathogens raises a major challenge in clinical practice, driving the development of antibacterial materials for biomedical applications. These advanced biomaterials aim to prevent microbial adhesion, inhibit biofilm formation, and reduce infection risk without compromising biocompatibility or mechanical performance. Strategies to confer antimicrobial activity include the incorporation of metal ions or nanoparticles (such as silver, copper, or zinc), the functionalization with antimicrobial peptides or natural extracts, and the design of smart, stimuli-responsive surfaces that can release antibacterial agents in response to infection-related cues. Recent research also emphasizes the balance between long-term antibacterial effectiveness and cytocompatibility to ensure safe integration with host tissues. Such innovations hold significant potential for improving the longevity and success of medical implants, wound dressings, and tissue engineering scaffolds [1,2,3]. Building upon these antibacterial strategies, interest has grown in metal–polymer nanocomposites (MPNs) due to their ability to synergize the potent antimicrobial effects of nanoscale metal particles, such as silver and copper, with the mechanical flexibility and processability provided by polymer matrices. This combination not only enhances the antimicrobial efficacy through sustained metal ions release and prevention of bacterial adhesion, but also allows for tailored mechanical properties and manufacturability essential for biomedical use [4]. As a result, MPNs have emerged as versatile functional materials with expanding applications [5]. Their growing interest arises from the advantageous physicochemical properties of nanoscale metals combined with the flexibility and shaping abilities of polymers [6,7]. This synergy enables the development of functional materials applicable in diverse fields, including optics [8], catalysis [9,10], and biomedicine [11,12,13]. Numerous research studies have demonstrated that integrating AgNPs into dental devices offers significant antimicrobial benefits. These tiny particles have demonstrated a significant ability to suppress bacterial proliferation within the oral environment, making them a valuable addition to various dental materials [14,15]. On the other hand, several methods have been reported for producing MPNs [6]. Solution-based approaches involve either incorporating preformed nanoparticles during polymer synthesis or post-reduction of previously dispersed metal salts within a polymer matrix using an external reducing agent. Alternatively, fabrication methods predicated on physical processes, such as mechanical incorporation, vapor phase deposition, or ion implantation, may be explored. Depending on the specific applications, precise control over parameters including metal loading, filling profile, and morphology of the metal particles is paramount. The current study investigates the development and characterization of PDMS/AgNPs nanocomposites, with a focus on examining their optical properties and biological behavior, aiming to combine two materials with distinct and complementary features. This functionalized PDMS-based nanocomposite embedded with silver AgNPs is designed as an antibacterial coating for optical fibers, with the primary aim of enhancing fiber protection in the field of photobiomodulation [16,17,18] while also improving patient comfort during phototherapeutic treatments using medical devices based on optical fibers. PDMS is widely favored across numerous applications due to its exceptional moldability, optical transparency, outstanding biocompatibility, and superior mechanical performance [19,20,21]. By incorporating AgNPs into the PDMS matrix, the resulting nanocomposite effectively combines optical clarity with robust antibacterial functionality. The inherent transparency and flexibility of PDMS ensure minimal disruption of light transmission, critical for maintaining the therapeutic efficacy of photobiomodulation. Meanwhile, the localized surface plasmon resonance properties of the embedded AgNPs may contribute to enhancing photonic interactions at the fiber-coating interface. Additionally, the potent antibacterial activity of AgNPs prevents biofilm formation on the fiber surfaces, thus safeguarding device integrity and reducing infection risks. This dual functionality is essential not only for prolonging optical fiber durability but also for ensuring patient safety and comfort during repeated or prolonged phototherapeutic applications [22,23]. Beyond its optical and antibacterial functionalities when combined with AgNPs, PDMS also stands out due to its unique physicochemical profile, including its elasticity, chemical inertness, thermal stability, and electrical/thermal insulating capacity, which establishes it as a material of choice in both scientific and industrial fields [19,21]. Within the biomedical sector, PDMS is routinely used for the fabrication of microfluidic devices, biomedical models, and medical implants, thanks to its tissue compatibility and its ability to minimize inflammatory responses upon implantation [14]. Furthermore, its transparency and capacity for fine structural replication make it highly valuable for integration into optical and microanalytical systems [16]. PDMS-based materials incorporating AgNPs primarily leverage the distinct optical and medical [24,25,26,27] properties of AgNPs. Conventional solution-based synthesis remains the standard methodology for preparing such composites [23,28,29]. However, an exclusive technique specifically tailored for PDMS was introduced by Goyal et al., involving in situ formation of AgNPs within the polymer matrix by mixing silver precursors with elastomers, followed by chemical reduction [30]. This method bypasses the need for preformed nanoparticles and obviates the requirement for external reducing or stabilizing agents. This procedure involves the incorporation of mixing a metal salt during the blending of the siloxane elastomer and its hardener. The Si-H silane function on the hardener serves a dual role, facilitating both the hydrosilylation reaction and the reduction in the silver salt to form nanoparticles. While this method yields samples with a highly homogeneous nanoparticle distribution, it may entail significant costs associated with metal salt, especially when producing materials on a large scale.

In this study, we introduce a method focused on the diffusion and in situ reduction of silver salt, resulting in the formation of small silver nanoparticles with a diameter of 10 nm within a preformed PDMS Sylgard 184-type polymer matrix. This simple and cost-effective approach, already reported for other applications [31], enables the rapid development of a nanocomposite with antibacterial properties, using a well-established PDMS-type polymer. Therefore, the purpose of the current study was to assess the effect of this elaborated coating on human gingival fibroblast behavior. Two hypotheses were suggested: (1) AgNPs would enhance gingival fibroblast cell behavior (proliferation and mineralization ability); (2) the incorporation of AgNPs into an experimental PDMS Sylgard 184-type polymer would affect its antibacterial activity.

## 2. Materials and Methods

Materials. PDMS Sylgard 184 was procured from Samaro (French distributor of Dow^®^, 01700 Beynost, France). Absolute ethanol, tetrahydrofuran (THF), and Silver tetrafluoroborate (AgBF_4_) were obtained from Sigma-Aldrich (38070 Saint-Quentin-Fallavier, France) and used without further purification. All glassware was cleaned with ethanol and air washed. Polypropylene cans were used as molds for sample preparation without modification.

Sylgard 184 reticulation. Sylgard 184 is a two-part commercial PDMS with an original ratio of 10:1 (Part A:Part B). Part A mainly comprises the vinyl-terminated polymer base along with the platinum catalyst. Part B contains the silane (Si–H) terminated cross-linker. Sylgard 184 PDMS undergoes cross-linking via the hydrosilylation process facilitated by a platinum catalyst. Briefly, the vinyl functions within the polymer base (part A) react with the silane functions on the cross-linker. The PDMS was mixed in a 10:1 ratio and cured at 70 °C for 2 h. After cross-linking and cooling, samples of 225.1 cm were cut before silver nanoparticle doping. For biocompatibility tests, samples were cut as disks of 14 mm radius to fit inside the wells of 24-well plates. The silanes contained in Sylgard 184-part B serve as the reactive functionalities of interest. During the crosslinking process, not all silanes may be linked, and thus some could be trapped inside the intricate chains within the polymer matrix. To the best of the authors’ knowledge, under appropriate conditions, these residual functionalities could undergo further reactions. Specifically, alkoxy (Si–OR) and silanol (Si–OH) groups are capable of reacting with water or alcohols through hydrolysis or transesterification, and can also undergo additional condensation with other silanol groups. Furthermore, depending on their chemical structure, these silane functionalities can react with nucleophilic species such as amines. These secondary reactions may influence the final structure, interfacial chemistry, and properties of the material [32].

Silver Nanoparticles Synthesis: To modify the PDMS, a dipping solution containing silver tetrafluoroborate was prepared. In brief, AgBF_4_ was dissolved in tetrahydrofuran (THF) to achieve a silver ion (Ag^+^) concentration of 0.1 M and magnetically agitated until a homogeneous solution was attained. The concentration of the Ag^+^ solution was determined through argentometric titration. Samples of Sylgard 184 PDMS were immersed in the solution for varying durations, ranging from 1 s to several hours. Subsequently, the samples were rinsed with ethanol, wiped with absorbent paper, and dried for 1 h in an oven at 70 °C.

Thermogravimetric analysis (TGA) and Differential Scanning Calorimetry (DSC): TGA and DSC measurements were realized on a TGA/DSC 3+ (Mettler-Toledo, 78220 Viroflay, France) and a DSC 1 (Mettler-Toledo), respectively. Thermogravimetric analyses were carried out to determine the thermal decomposition profiles of the tested samples, with or without AgNPs. The samples were placed in 150 µL alumina crucibles and heated from 30 °C to 850 °C under pure air at a heating rate of 10 K·min^−1^. The TGA data were plotted as weight (%) versus temperature (°C) to assess the thermal behavior of the tested materials. For DSC analyses, samples were heated in 40 µL aluminum crucibles from −150 °C to 50 °C with a heating rate of 5 K·min^−1^, under pure nitrogen, in order to measure the glass transition (Tg) of samples.

Cell Culture: Human gingival fibroblast (HGF) cells were collected from gingival tissue biopsies of patients undergoing orthodontic extractions, in accordance with the French legislation. Cells were then utilized at passage 3, and cultures were maintained at 37 °C in a humidified atmosphere containing 5% CO_2_ in air. The culture medium was refreshed every 2 days. Cells were cultured in Dulbecco’s modified Eagle medium (DMEM) (11995073, Gibco™, Villebon-sur-Yvette, France) supplemented with 10% fetal bovine serum (FBS) (10500064, Gibco™, France), 1% Penicillin/Streptomycin and 0.2% Amphotericin B (#0513 and #0523, ScienCell, Carlsbad, CA, USA). For the current study, 24-well culture plates were utilized. The cells were directly seeded onto the sample surfaces at a concentration of 2 × 10^4^ cells per sample. A total of 500 μL of medium was added to the wells containing the samples, resulting in a total media suspension of 1 mL per well.

Alamar Blue assay: The metabolic activity of HGF cells was evaluated with the Alamar Blue assay at 1, 3, and 7 days after contact with the sample surfaces. Each sample type was tested in quadruplicate in three independent experiments (n = 12) using 24-well plates, following standard cytocompatibility testing practices consistent with ISO 10993-5 recommendations to ensure sufficient statistical reliability and reproducibility. Briefly, 200 μL of the culture medium from each well was aspirated twice and transferred to a 96-well plate, yielding duplicate measurements per sample well. Plates were incubated for 8 h at 37 °C in a humidified atmosphere with 5% CO_2_ before analysis. Alamar Blue is blue in its oxidized form and changes to pink or purple when reduced to resorufin by metabolically active cells. Resorufin levels were determined spectrometrically at 570 nm and 600 nm using a microplate reader (Infinite^®^ M200 PRO NanoQuant, Tecan Group Ltd., Lyon, France). Results were expressed as a percentage of cell viability compared to the untreated control (100%).

Cell morphology: Fluorescence microscopy (Olympus CKX41 microscope, Rungis, France) was employed to observe the cells following staining of actin and nuclei. Fluorescence staining was conducted to visualize morphological changes. Cells were washed three times with Phosphate-Buffered Saline (PBS) (D8537, Gibco, Villebon-sur-Yvette, France). Subsequently, they were fixed for 30 min by incubation in 3.7% formaldehyde in PBS (10426730, Thermo Fisher Scientific, Villebon-sur-Yvette, France), followed by further washing. The cells were permeabilized with 0.1% Triton X100 (X100, Sigma Aldrich, Villebon-sur-Yvette, France) in PBS and then blocked with 1% bovine serum albumin (354331, Corning, Villebon-sur-Yvette, France) in PBS. Cell membranes were stained with AlexaFluor^®^ 488 phalloidin (A12379, Invitrogen, Villebon-sur-Yvette, France) at room temperature for green fluorescence. Cell nuclei were stained using DAPI (4′,6-diamidino-2-phenylindole D1306, Invitrogen, Villebon-sur-Yvette, France) at room temperature to visualize nuclei (blue fluorescence). Acquisitions were collected sequentially (green fluorescence/blue fluorescence) and then merged.

Antibacterial activity assessment: To conduct the antibacterial test, *Escherichia coli* (ATCC 25922, Institut Pasteur, Paris, France) was selected as the bacterial strain. Following culturing of the bacteria under aerobic conditions, the tested samples (pure Sylgard 184 samples as well as Sylgard 184 doped with low and high concentrations of AgNPs) were placed in agar Petri dishes. Freshly formed *E. coli* culture was spread onto the sample surfaces on the agar plates. The Petri dishes were then incubated at 37 °C for 72 h. To observe adhering biofilm, samples were fixed in 3.7% formaldehyde (*v/v*) in PBS for 1 h at room temperature, rinsed once with PBS, and dried in a graded series of ethanol solutions (25–100%). Samples were sputter-coated to achieve a 10 nm copper layer and then examined by field emission scanning electron microscopy (SEM) under a voltage of 5 kV using the FEI-Quanta 250 instrument from Thermo Fisher Scientific, Villebon-sur-Yvette, France.

Statistical analysis: The software SPSS TM (SPSS TM Statistics v23; IBM Corp, Bloomington, IL, USA) was used for all statistical analyses. One-way analysis of variance (ANOVA) followed by Games-Howell Post Hoc tests to investigate differences in metabolic activity (Alamar Blue assay = Results are reported as mean standard deviation (±SD)) and statistical significance was accepted as * *p* < 0.05 and ** *p* < 0.001.

## 3. Results and Discussion

The ability of PDMS to behave like a sponge upon contact with certain solvents allows consideration of solute diffusion into the polymer network during swelling. However, the choice of solvent/solute pair is crucial. For instance, the polarity and the solvodynamic size of a molecule strongly influence its capacity to penetrate the PDMS network [33]. Among the various solvent/solute pairs tested, the THF/AgBF_4_ pair proved to be the most suitable for the current purpose. THF is one of the best solvents for swelling PDMS [34]. Silver tetrafluoroborate is highly soluble in polar solvents such as THF (tetrahydrofuran), which facilitates its use in various organic and organometallic reactions. The silver ion (Ag^+^), paired with the tetrafluoroborate anion (BF_4_^−^), serves as a moderately strong oxidant and is particularly effective in promoting reduction reactions due to the weakly coordinating nature of the tetrafluoroborate ion. This property is especially useful for replacing halide ligands and for reactions requiring efficient electron transfer [35,36]. PDMS/silver nanoparticle nanocomposites were prepared by immersing PDMS samples (Sylgard 184, 10:1 ratio) in a 0.1 M solution of silver tetrafluoroborate (AgBF_4_) in THF. After immersion, the samples were briefly rinsed with ethanol and subsequently heated in an oven at 70 °C for 1 h. This thermal treatment is required to promote the reduction of silver ions. The concentration of AgNPs incorporated into the PDMS matrix depends on the immersion time in the silver precursor solution.

**Figure 1:**
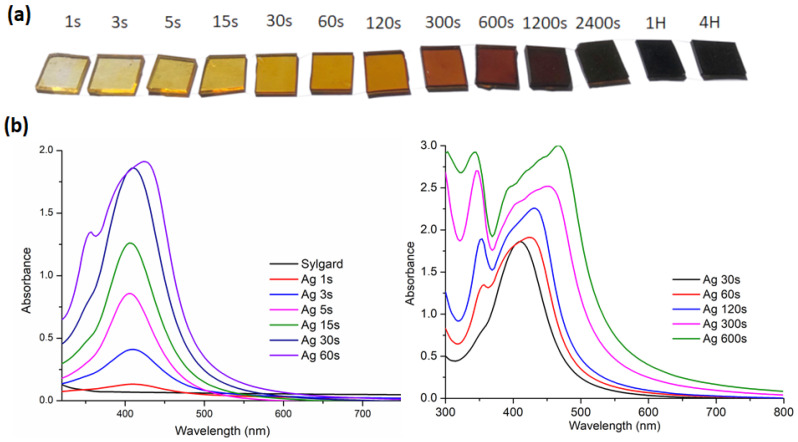
(**a**) Photographs of Sylgard 184 incorporating silver nanoparticles at increasing immersion durations in the silver solution; (**b**) UV–visible absorption spectra of Sylgard 184 after immersion in the silver solution for varying times.

**Figure 2:**
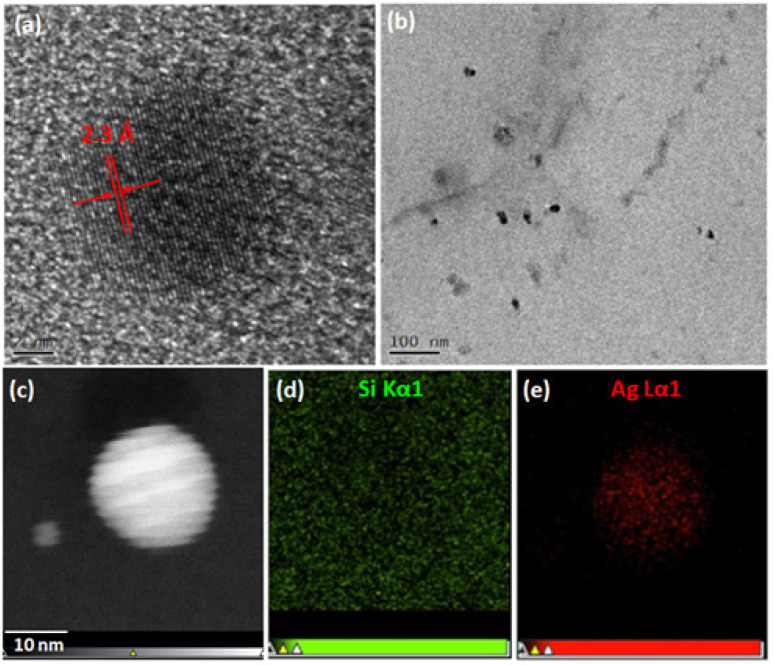
TEM characterization of Ag60s. (**a**) High-resolution TEM image showing the (111) lattice planes of a monocrystalline particle; (**b**) low-resolution TEM image; (**c**) STEM image of a crystalline particle; (**d**,**e**) Si and Ag elemental maps from the Si Kα1 and Ag Lα1 signals (STEM–EDX).

**Figure 3:**
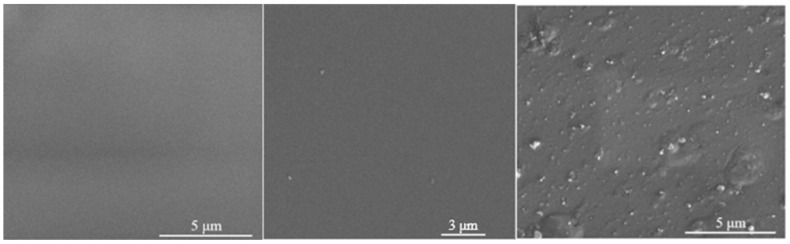
SEM images of the sample surfaces: (**left**) pure Sylgard 184, (**middle**) Ag60s, and (**right**) Ag1H.

The different samples prepared are named according to their immersion times: Ag5s, Ag15s, Ag30s, Ag1min, up to Ag4H. Figure 1a shows a photograph of Sylgard 184 pieces soaked for different times. From 1 s of soaking, the samples exhibit a yellow tint that increases with soaking time. All samples show absorption spectra characteristic of the presence of AgNPs (Figure 1b). The spectra for samples ranging from Ag1s to Ag30s exhibit an absorption band localized at 410 nm, characteristic of the absorption of spherical silver nanoparticles with a diameter of approximately 10 nm in a silicate environment [37]. The nanoparticle size was confirmed by TEM microscopy, as shown in Figure 2. For these samples, the presence of a single narrow absorption band indicated good monodispersity and the absence of nanoparticle agglomeration within the polymer network. In Ag5s, Ag15s, and Ag30s, the absorbance increased linearly with soaking time. For soaking times longer than 30 s, the spectra evolved, displaying two distinct absorption bands likely associated with multimodal resonance [38]. Moreover, Figure 2b reveals numerous short-range interactions between pairs of silver particles. This phenomenon was attributed to nanoparticle interactions and was consistent with an increase in nanoparticle concentration within the PDMS matrix. After an extended soaking time, the concentration of AgNPs increased, inevitably promoting their mutual interactions. This was due to the gradual saturation of free sites in the siloxane network. Moreover, the SEM image (Figure 3) showed that from one hour onward, nanoparticle aggregates could be observed on the material’s surface.

**Figure 4:**
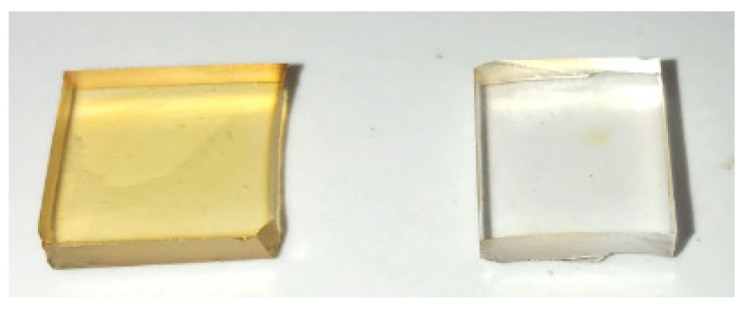
Sample Ag30s treated at 70 °C (**left**) and Ag30s stored at room temperature for 24 h (**right**).

Previous studies have reported that residual silane groups (Si–H) in PDMS can act as reducing agents for Ag^+^ ions [39,40]. An instantaneous reduction of silver ions occurs when the curing agent containing silane groups is introduced into a solution of AgBF_4_ in THF. In contrast, no reaction is observed when only the elastomer, lacking Si–H groups, is added to the silver solution, thereby confirming the essential role of silane groups in silver particle formation. The presence of residual Si–H silane groups in the 10:1 Sylgard polymer prior to immersion was confirmed by solid-state ^29^Si NMR. The fraction of Si–H groups in the pristine polymer was estimated at 1.7%. A slight decrease was observed in the Ag15s and Ag60s samples, to 1.1% and 1.2%, respectively, with no significant difference between these two samples. Thermal activation was necessary for silver ion reduction (Figure 4). No yellow coloration, indicative of silver nanoparticle formation, was observed for the Ag15s sample maintained at room temperature, in contrast to the sample treated at 70 °C. These results indicate that while Si–H groups are essential for silver particle formation, thermal activation is also required [41,42]. The absence of reactivity at room temperature suggests the presence of a kinetic barrier associated with the activation of Si–H groups. Increasing the temperature to 70 °C enhances both the mobility of the siloxane chains and the reactivity of Si–H bonds, resulting in the incorporation of silver species into the network. This indicates that thermal activation plays a key role in overcoming the energy barrier that limits the reaction under ambient conditions.

The SEM images in Figure 3 compare the surfaces of pure Sylgard 184, Ag60s, and Ag1H samples. The Sylgard 184 surface was smooth and homogeneous, as expected for pristine PDMS. The Ag60s sample also showed a relatively homogeneous morphology, with some unidentified particles present. In contrast, the Ag1H surface was densely covered with particles, either agglomerated or dispersed, and numerous small surface protrusions were observed, indicating degradation of the PDMS matrix.

The formation of crystalline silver nanoparticles was confirmed by HR-TEM analysis of a microtome section of the Ag1H sample (Figure 2a), where clear (111) lattice fringes were observed. Figure 2b shows a homogeneous distribution of nanoparticles with an average diameter of approximately 10 nm. The incorporation of nanoparticles into the PDMS matrix was further confirmed by scanning transmission electron microscopy (STEM) and elemental mapping by EDS (Figure 2c–e).

**Figure 5:**
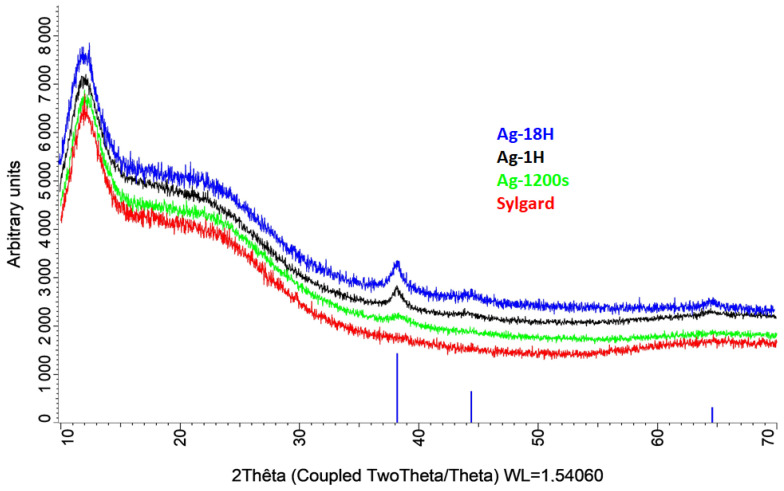
X-ray diffraction (XRD) patterns of Ag1200s (green), Ag1H (black), and Ag18H (blue). The blue bars indicate the reference XRD pattern of silver (JCPDS #00-004-0783).

X-ray diffraction analysis (Figure 5) revealed that diffraction peaks corresponding to the (111) lattice plane of silver (space group Fm-3m) appear only after a soaking time of 20 min. For longer soaking times (e.g., Ag18H), additional peaks corresponding to the (200) and (220) planes were observed. Regardless of soaking duration, the size of the silver monocrystals, estimated from the full width at half maximum (FWHM) of the (111) peak, was approximately 10 nm. This value is consistent with the HR-TEM analysis (Figure 2a).

**Figure 6:**
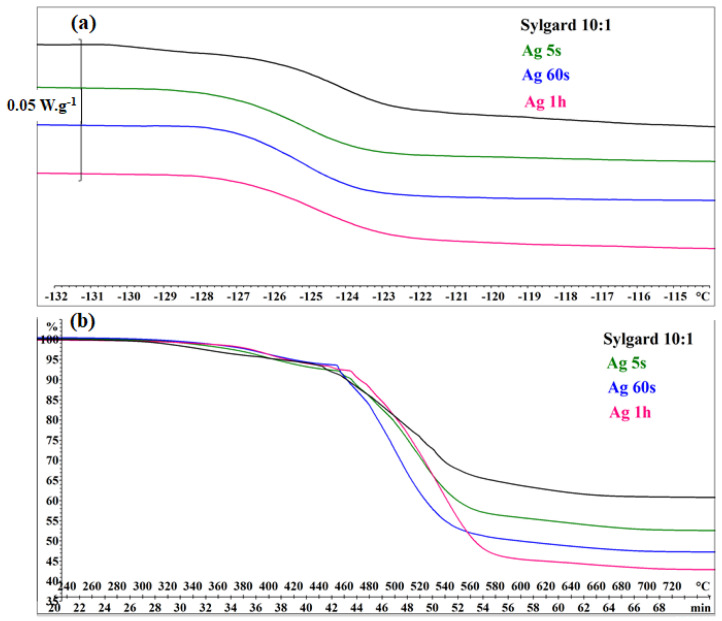
(**a**) DSC curves and (**b**) TGA curves of SYL10:1 (black), Ag5s (green), Ag60s (blue), and Ag1H (red).

DSC and TGA analyses (Figure 6) were performed on pure Sylgard 10:1 as well as on Ag5s, Ag60s, and Ag1H samples. All DSC curves exhibited a glass transition at −126 °C (Midpoint). The TGA curves indicated a higher thermal stability limit compared to commercial Sylgard 184, which begins to decompose at 301 °C. An increase in silver content led to a greater mass loss during degradation. The total mass loss (Δmi/mi, where mi is the initial mass) was 39.6% for pure Sylgard 10:1, 47.7% for Ag15s, 53.3% for Ag60s, and 57.3% for Ag1H. These results suggest that the presence of silver nanoparticles promotes intra- and intermolecular rearrangements toward more volatile cyclic oligomers during thermal degradation [43].

- **Cytocompatibility assessment**
- Cell metabolic activity via Alamar Blue assay (AB)

**Figure 7:**
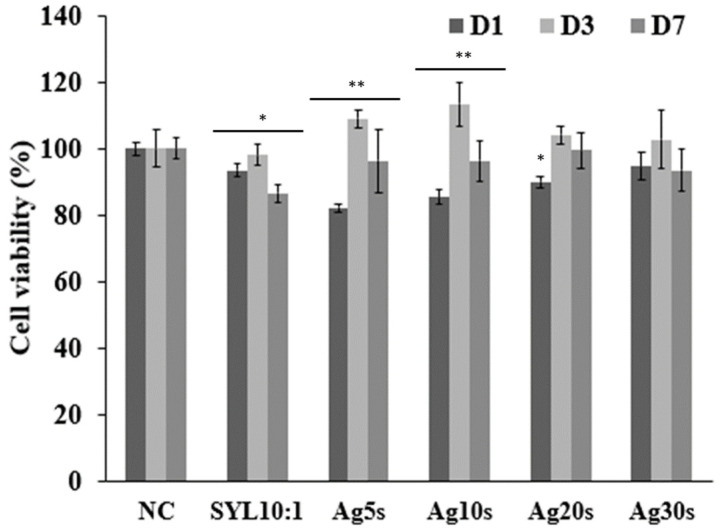
Cell viability of pure Sylgard 184 and Sylgard 184 samples immersed in the silver solution for different durations, compared to the negative control. Data are presented as mean ± SD of 3 independent experiments (n = 9). * *p* < 0.05 and ** *p* < 0.001 indicate statistically significant differences.

The histograms in Figure 7 present a comparison of cell viability, assessed via cell metabolic activity, between the negative control (NC = 100%) and the tested samples after 1, 3, and 7 days of contact with the cells. All samples demonstrated excellent cell viability over time. Initially, all samples, including pure Sylgard 184 PDMS, exhibited slightly lower viability than the negative control, yet values remained high, between 80 and 90%. After 3 days, all samples containing AgNPs, regardless of concentration, showed higher viability than both Sylgard 184 and the NI, with percentages exceeding 100%. Prolonged contact with AgNP-doped samples further enhanced cell activity, while Sylgard 184 alone still achieved 98% viability. By day 7, all samples exhibited a slight decline in intrinsic cell viability. The Ag10s sample showed the largest decrease (from 125% to 95%), whereas Ag30s showed the smallest decrease (from 102% to 93%). Nevertheless, all samples maintained high cell viability, around 90%. Pure Sylgard 184 exhibited slightly lower viability. The most consistent performance over the entire experiment was observed for pure Sylgard 184, Ag20s, and Ag30s, whereas Ag5s and Ag10s showed greater variability.

The cytotoxicity of AgNPs is primarily attributed to the release of free Ag^+^ ions from the nanoparticles, and the level of toxicity depends on the concentration of these ions in solution [44,45]. As seen in Figure 3, most of the nanoparticles are embedded within the polymer, with only a few present on the surface of the samples. Therefore, the initial release of Ag^+^ ions into the culture medium after 24 h of contact with the cells likely explains the reduced initial cell viability. During the subsequent rinsing steps and changes in the culture medium between measurements, the free silver ions were removed, preventing further release from the embedded nanoparticles.

For the subsequent assays, only Ag5s and Ag30s samples were used for biocompatibility tests, selected to represent the lowest and highest concentrations of AgNPs in Sylgard 184. ICP-MS measurements were performed on three types of samples, Sylgard 184, Ag5s, and Ag3s, to evaluate the release of silver ions in water at these minimum and maximum AgNP concentrations. The results are summarized in Table 1.

ICP–MS measurements indicated that no Ag^+^ ions were released from the Sylgard 184 sample. For the Ag5s and Ag30s samples, the Ag^+^ concentrations were similar, measuring 1.3 and 1.2 µg/L, respectively, after 1 h of immersion. After 6 h in deionized water, the Ag^+^ concentration decreased by approximately half (0.67 µg/L), and no Ag^+^ ions were detected after 24 h. These results suggest that Sylgard 184 samples containing AgNPs undergo a rapid release of silver ions in water, with the process being essentially complete within 24 h.

These findings also provide an explanation for the cell viability results shown in Figure 7. The first Alamer Blue (AB) quantification was performed after 24 h without prior rinsing, resulting in the highest Ag^+^ concentration in the culture medium, which could explain the initially reduced cell viability. After this period, no Ag^+^ ions were detected in the solution, allowing the cells to proliferate in the absence of free silver ions. These results are consistent with previous studies reporting that the rapid release of Ag^+^ from nanoparticles can transiently decrease cell viability as measured by the Alamar Blue assay [46,47]. In the current study, the in vitro release of Ag^+^ ions from the samples was evaluated using ICP–MS measurements, and the resulting effects on cell viability were discussed. The data showed a rapid initial release of silver ions within the first 24 h, followed by negligible release thereafter, which correlates with the transient decrease in cell viability. While these findings provide valuable insights into the early-stage behavior of AgNPs in vitro, further studies are needed to investigate long-term release profiles. Such studies could include both extended in vitro experiments and in vivo evaluations, focusing on the stability of AgNPs under physiological conditions, cumulative ion release over time, and interactions with biological media. These investigations would be particularly relevant for assessing the safety and efficacy of AgNP-containing materials in clinical applications, especially for prolonged exposure scenarios.

- **Cell morphology**

**Figure 8:**
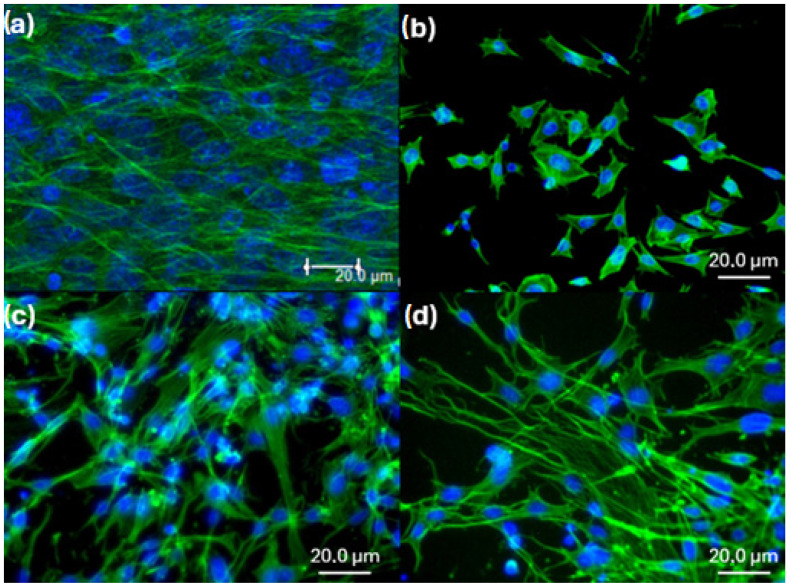
Representative confocal fluorescence images of human gingival fibroblast (HGF) cells adhered on (**a**) negative control (glass slide), (**b**) Sylgard 184, (**c**) Ag5s, and (**d**) Ag30s surfaces after 7 days of culture. The cytoskeletons are stained green and the nuclei blue.

Confocal images of HGF cells after 7 days of culture are shown in Figure 8. The cytoskeletons are stained green, while the nuclei appear blue. In the negative control (Figure 8a), cells proliferated to near confluence and exhibited well-stretched morphologies, confirming strong adhesion. On Sylgard 184 (Figure 8b), fewer cells adhered compared to the control; however, those present displayed well-spread shapes, indicating good attachment. The AgNP-containing samples (Figure 8c,d) showed higher cell densities than pure PDMS but lower than the control. In both cases, HGF cells exhibited elongated cytoskeletal fibers and a well-spread morphology, suggesting strong adhesion. These observations are consistent with the Alamar Blue assay, supporting that all tested samples are cytocompatible and allow cell adhesion and proliferation.

- **Live/Dead staining**

**Figure 9:**
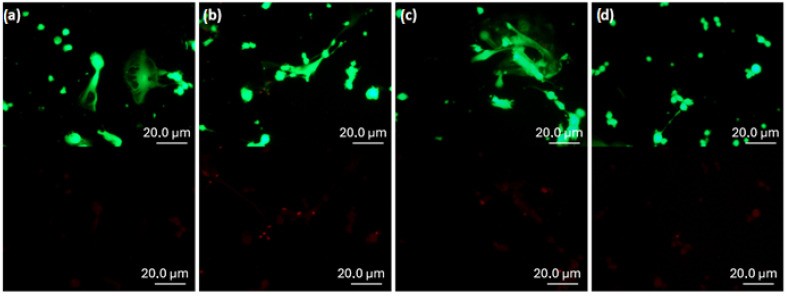
Fluorescence images of human gingival fibroblasts (HGFs). Viable cells are stained with calcein-AM (green, top row), while damaged cells are stained with EthD-1 (red, bottom row). (**a**) Negative control (glass slide), (**b**) Sylgard 184, (**c**) Ag5s, and (**d**) Ag30s.

To further evaluate the cytotoxicity of the samples, a Live/Dead staining assay was performed after 24 h of incubation. Representative fluorescence images of the tested samples are shown in Figure 9.

Green fluorescence was consistently observed across all samples, while red signals were negligible and likely attributable to imaging artifacts caused by color saturation. These results indicate that the vast majority of cells remained viable, confirming the absence of cytotoxic effects in contact with the tested nanocomposites.

- **Antibacterial activity**
- Antibiograms assay and samples bacterial colonization

**Figure 10:**
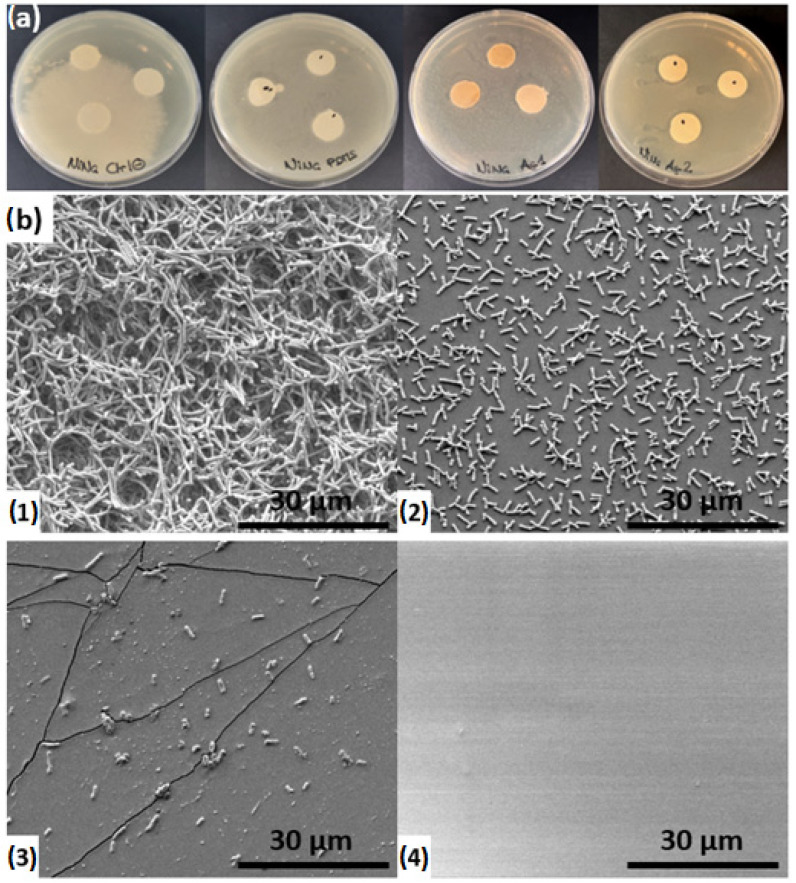
(**a**) Photograph of antibiogram assays for the negative control, Sylgard 184, Ag5s, and Ag30s samples (from left to right). (**b**) Representative SEM images showing *E. coli* colonization on the surface of (**b1**) negative control, (**b2**) Sylgard 184, (**b3**) Ag5s, and (**b4**) Ag30s samples.

Antibiogram tests were performed on three Sylgard 184 samples (pure, Ag5s, and Ag30s) and were compared to a control. Figure 10a shows the bacterial spreading after 72 h. Quantitative measurements were not possible due to the inhomogeneous bacterial distribution in the gel, which made it impossible to determine halo diameters. Nevertheless, a qualitative analysis was feasible. Control samples exhibited extensive bacterial colonization, while pure Sylgard 184 samples displayed a small surrounding halo. AgNP-loaded Sylgard 184 samples (Ag5s and Ag30s) showed little to no halo, indicating effective inhibition of bacterial colonization, which corroborates observations in similar studies on PDMS/silver nanoparticle composites [28,48].

Furthermore, Sharma et al. demonstrated an anticancer activity of AgNPs regarding A549 cells as revealed by the Alamar Blue assay, without reporting antibacterial effects [47]. This action highlights the diverse biofunctional activities of AgNPs according to the investigated biological system.

- **Biofilm observation**

SEM images of the four samples were obtained after fixation of bacterial colonization, as shown in Figure 10b. The surface of the control sample (Figure 10(b1)) was completely covered with bacteria, forming thick multilayered colonization. The underlying substrate was not visible, as the high density of *E. coli* bacterial cells formed a biofilm on the surface. The Sylgard 184 sample (Figure 10(b2)) also exhibited bacterial colonization, with cells distributed homogeneously across the surface; however, the bacterial density was lower, and the substrate surface remained partially visible. The Ag5s sample (Figure 10(b3)) displayed only a few bacteria on its surface, while the Ag30s sample (Figure 10(b4)) appeared entirely free of bacterial colonization.

The bacterial biofilm formed on the control sample can be linked to the bacterial halo visible on Figure 10a. Sylgard 184 is hydrophobic, with an average contact angle of approximately 110° [19]. Its inherent hydrophobic surface may explain the low bacterial colonization observed, as hydrophobicity can act as an antifouling mechanism. The reduced to absent presence of *E. coli* on the Ag5s and Ag30s samples could therefore be partly attributed to the same surface property. However, an additional mechanism is potentially involved. Silver nanoparticles and silver ions exert bactericidal effects at different levels, with a synergisticactivity [36,49]. As shown in Table 1 only a small amount of silver ions was released from our samples. Therefore, the antibacterial effect could be attributed primarily to the AgNPs themselves. W. Li et al. [50] suggested that AgNPs induced an inactive state of the respiratory chain hydrogenase. disrupts membrane permeability and ultimately leads to the death of *E. coli* cells.

On the other hand, the bactericidal activity of AgNPs is highly dependent on both their size and concentration, whether dispersed in solution or immobilized on a surface. Numerous studies have shown that smaller AgNPs, particularly those with diameters near 10 nm, exert significantly stronger antibacterial effects than larger particles, owing to their higher surface area and reactivity [51,52,53]. Ershov et al. further established a clear linear correlation between antibacterial activity and nanoparticle size, showing that smaller particles of 10.8 ± 0.8 nm display enhanced toxicity against Escherichia coli and other bacteria. This enhanced antibacterial effect is directly linked to the increased specific surface area, which facilitates greater silver ion release and improved bacterial cell penetration [54]. In this study, AgNPs of approximately 10 nm in diameter were synthesized, which, based on the literature, should exhibit pronounced bactericidal activity. However, a limited distribution of AgNPs on the surface of samples was observed, which appears to induce a predominantly bacteriostatic rather than a fully bactericidal effect, underscoring the importance of surface coverage in addition to particle size.

The present research study demonstrated how PDMS can be leveraged as a tunable matrix for the synthesis and incorporation of AgNPs, exploiting its sponge-like swelling properties in specific solvents such as THF. The choice of the solvent/solute pair was found to be critical for optimized nanoparticle formation, as the polarity and solvodynamic size of both solvent and solute dictate the efficiency of diffusion into the polymer network. Consistent with previous findings [33], THF proved to be one of the most effective solvents for swelling PDMS and allowing efficient incorporation of silver tetrafluoroborate.

Nanocomposites were prepared by immersion in AgBF_4_/THF solution, with systematic analysis revealing that soaking duration strongly influences both nanoparticle loading and their optical features. A progressive increase in soaking time resulted in a distinct yellowing of samples, a linear growth in absorbance at 410 nm (indicative of spherical AgNPs with a diameter of ~10 nm), and a shift to multimodal resonance at longer immersion, corroborating nanoparticle interaction and agglomeration phenomena [41,42]. These phenomena were confirmed by HR-TEM and STEM/EDS analyses, which evidenced crystalline AgNPs homogeneously distributed within the PDMS matrix at the mesoscale.

Thermogravimetric analysis indicated that silver incorporation accelerates mass loss during thermal degradation, possibly through the promotion of cyclization and formation of more volatile cyclic oligomers [44]. Biocompatibility assays revealed high cell viability for all AgNP-loaded and pure PDMS samples, in line with literature on PDMS and AgNP cytocompatibility and their limited ion release after the initial hours post-immersion. ICP-MS showed only short-term release of Ag^+^ ions, consistent with initial cytotoxicity in the alamarBlue assay and recovery thereafter.

Antibacterial assays, including qualitative antibiograms and SEM imaging, highlighted significant inhibition of *E. coli* colonization for AgNP-loaded samples, whereas pure Sylgard 184 and controls showed substantially more biofilm formation [45,46]. The hydrophobic and antifouling nature of PDMS may partially explain the reduced colonization, but the primary effect appears to be attributable to the presence of AgNPs and, at early time points, to Ag^+^ ions [48,49,55]. The bactericidal action is also dependent on both nanoparticle size and spatial distribution. As confirmed in recent studies, smaller AgNPs (~10 nm) show significantly stronger antibacterial activity due to their higher surface area and reactivity, but sparse distribution at the surface of polymer samples may limit efficacy to a bacteriostatic effect rather than full bactericidal inhibition [28,52].

In summary, our findings reinforce the importance of solvent-polymer dynamics, nanoparticle synthesis parameters, and size/concentration effects in dictating the biocompatibility and antibacterial performance of AgNP–PDMS composites. This understanding provides key insights for designing next-generation biomimetic materials with tailored antimicrobial and cytocompatible properties.

## 4. Conclusions

In this study, we have developed a simple, rapid, and cost-effective strategy to produce a silver-based metal–polymer nanocomposite by in situ reduction of silver ions within Sylgard 184 PDMS. The process yields well-distributed AgNPs (~10 nm), which impart a distinct yellow coloration while preserving the intrinsic properties of PDMS. The resulting nanocomposites exhibit both enhanced antibacterial activity and good cytocompatibility, overcoming one of the main limitations of conventional AgNP systems. Owing to the ease of controlling AgNP concentration and distribution, this material represents a promising platform for designing non-toxic antibacterial coatings with potential applications in a wide range of biomedical applications. Future investigations into the release mechanisms and long-term stability of these composites could further enable their applicability in areas such as phototherapy and implantable medical devices.

## Figures and Tables

**Table 1:** Concentrations of silver ions released from Sylgard 184, Ag5s, and Ag30s samples in deionized water (dH_2_O) over time.

	[Ag^+^] (µg/L) After 1 h	[Ag^+^] (µg/L) 6 h	[Ag^+^] (µg/L) 24 h	[Ag^+^] (µg/L) 72 h
Sylgard 184	<0.1	<0.1	<0.1	<0.1
Ag5s	1.3	0.67	<0.1	<0.1
Ag30s	1.2	0.67	<0.1	<0.1

## Data Availability

All the data of the current study are provided in the main manuscript. The raw data can be provided by the authors if needed.
